# Supplementary material for: The Protective Role of Exosome-Derived MicroRNAs and Proteins from Human Breast Milk against Infectious Agents
Source: Metabolites. 2023 May 8;13(5):635. doi: 10.3390/metabo13050635 (PMC10221093; doi:10.3390/metabo13050635)
Supplement: Supplementary file 1 [file metabolites-13-00635-s001.zip › metabolites-2362119-supplementary.pdf]

Table S1. Total GO annotation of HBM-derived exosomal miRNA

Term name	Number of related genes	Number of miRNA	P-value
Gene Ontology: Cell Component			
cytosol	943	17	< 0.05
nucleoplasm	425	19	< 0.05
protein complex	1261	19	< 0.05
cellular component	5978	30	< 0.05
organelle	4753	53	< 0.05
Gene Ontology: Molecular Function			
enzyme binding	516	20	< 0.05
nucleic acid binding transcription factor activity	470	26	< 0.05
protein binding transcription factor activity	228	28	< 0.05
molecular function	6721	43	< 0.05
ion binding	2929	52	< 0.05
HECT domain binding	3	17	3.3E-16
cytoskeletal protein binding	272	12	2.2E-15
enzyme regulator activity	224	7	3.3E-06
miRNA binding	7	9	2.9E-02
Gene Ontology: Biological Process			
biosynthetic process	1839	46	1.3E-28
gene expression	290	35	1.7E-11
immune response		34	2.8E-02
cell death	242	9	3.6E-06
mitotic cell cycle	72	6	1.1E-02
signal transduction	608	3	1.6E-04
cell adhesion	38	1	1.4E-03
cell differentiation	6	1	2.6E-02
cell proliferation	3	1	4.4E-02

Table S2. Immune-related GO annotation of HBM-derived exosomal miRNA

GO Category :Biological Process (immune related)	Number of related gene	Number of miRNA	P-value
Fc-epsilon receptor signaling pathway	102	34	1.79E-15
Fc-gamma receptor signaling pathway involved in phagocytosis	25	3	1.58E-09
toll-like receptor 10 signaling pathway	20	5	1.04E-04
toll-like receptor TLR1:TLR2 signaling pathway	20	5	1.71E-04
toll-like receptor TLR6:TLR2 signaling pathway	20	5	1.71E-04
toll-like receptor 5 signaling pathway	20	4	3.01E-04
TRIF-dependent toll-like receptor signaling pathway	22	6	3.85E-04
toll-like receptor 4 signaling pathway	28	5	4.85E-04
toll-like receptor signaling pathway	25	4	5.72E-04
toll-like receptor 9 signaling pathway	20	4	5.72E-04
MyD88-independent toll-like receptor signaling pathway	19	4	7.19E-04
toll-like receptor 2 signaling pathway	20	4	1.17E-03
toll-like receptor 3 signaling pathway	17	3	1.40E-03
stress-activated MAPK cascade	15	3	1.48E-03
activation of phospholipase C activity	13	2	2.04E-03
immune system process	132	2	4.85E-03
innate immune response	84	3	6.36E-03
JAK-STAT cascade involved in growth hormone signaling pathway	6	1	7.10E-03
intrinsic apoptotic signaling pathway	10	1	7.25E-03
MyD88-dependent toll-like receptor signaling pathway	20	3	7.33E-03
neuron cell-cell adhesion	8	3	9.00E-03
cellular response to glucagon stimulus	6	2	9.97E-03
apoptotic signaling pathway	22	2	1.09E-02
intracellular receptor signaling pathway	6	2	1.60E-02
positive regulation of cell proliferation in bone marrow	1	1	2.04E-02
negative regulation of interleukin-13 production	1	1	2.04E-02
T-helper 1 cell differentiation	1	1	2.40E-02
negative regulation of interleukin-5 production	1	1	2.40E-02
negative regulation of interleukin-4 production	1	1	2.40E-02
neutrophil differentiation	1	1	2.40E-02
negative regulation of transcription, DNA-templated	11	1	2.50E-02
positive regulation of granulocyte differentiation	1	1	2.67E-02
transforming growth factor beta receptor signaling pathway	17	2	3.22E-02
alpha-beta T cell differentiation	1	1	3.22E-02
inositol phosphate metabolic process	11	2	3.30E-02
positive regulation by host of viral transcription	1	1	3.41E-02
activation of protein kinase A activity	5	2	3.62E-02
positive regulation of signal transduction	10	1	4.10E-02
positive regulation of activated T cell proliferation	1	1	4.18E-02
cellular response to interleukin-4	1	1	4.18E-02
cellular response to cytokine stimulus	1	1	4.22E-02
regulation of Wnt signaling pathway	1	1	4.26E-02
negative regulation of peptidyl-threonine phosphorylation	1	1	4.41E-02
positive regulation of cytokine production	1	1	4.63E-02
positive regulation of epithelial to mesenchymal transition	1	1	4.86E-02

Table S3. Total KEGG pathway analysis of HBM-derived exosomal miRNA

Term name	Number of Related gene	Number of miRNA	P-value
Proteoglycans in cancer	118	36	1.76E-06
Glioma	38	32	1.56E-05
FoxO signaling pathway	75	27	2.20E-04
ECM-receptor interaction	42	25	6.57E-27
Signaling pathways regulating pluripotency of stem cells	67	24	6.92E-06
Pathways in cancer	154	23	7.98E-05
Focal adhesion	102	23	4.48E-04
TGF-beta signaling pathway	42	21	3.45E-06
Regulation of actin cytoskeleton	113	21	2.65E-04
Transcriptional misregulation in cancer	74	20	1.50E-03
Mucin type O-Glycan biosynthesis	14	19	8.93E-11
Wnt signaling pathway	50	19	9.33E-07
MAPK signaling pathway	111	19	5.85E-05
PI3K-Akt signaling pathway	149	19	3.45E-04
mTOR signaling pathway	30	19	1.86E-03
Estrogen signaling pathway	49	18	4.78E-06
Morphine addiction	44	17	1.31E-06
Renal cell carcinoma	36	17	1.53E-05
Glycosaminoglycan biosynthesis - keratan sulfate	10	17	2.22E-05
Dilated cardiomyopathy	34	17	4.45E-03
Dorso-ventral axis formation	14	17	5.44E-03
p53 signaling pathway	35	16	1.59E-05
Basal cell carcinoma	17	16	2.59E-04
Oocyte meiosis	36	16	7.68E-04
Non-small cell lung cancer	25	16	2.09E-03
Rap1 signaling pathway	105	16	7.11E-03
Cytokine-cytokine receptor interaction	51	15	8.73E-08
ErbB signaling pathway	49	15	5.50E-07
Amphetamine addiction	32	15	2.53E-06
Neurotrophin signaling pathway	63	15	8.33E-06
Melanoma	29	15	3.31E-05
Long-term depression	23	15	2.81E-04
Long-term potentiation	37	15	4.08E-04
Ras signaling pathway	101	15	9.41E-04
Hypertrophic cardiomyopathy (HCM)	37	15	1.93E-03
AMPK signaling pathway	51	15	4.36E-03
Hippo signaling pathway	64	14	4.95E-09
Lysine degradation	24	14	9.71E-07
Thyroid cancer	12	14	7.51E-05
Prostate cancer	45	14	4.32E-04
Ubiquitin mediated proteolysis	57	13	2.33E-05
Valine, leucine and isoleucine biosynthesis	1	13	6.16E-04
cGMP-PKG signaling pathway	66	13	1.96E-03
cAMP signaling pathway	82	13	8.15E-03
GABAergic synapse	32	12	2.46E-06
Amoebiasis	14	12	4.31E-06
Thyroid hormone signaling pathway	49	12	4.19E-05
Glycosaminoglycan biosynthesis - heparan sulfate / heparin	9	11	1.57E-08
Phosphatidylinositol signaling system	33	11	3.85E-06
Circadian entrainment	37	11	1.19E-05
Cocaine addiction	20	11	1.47E-04
Chronic myeloid leukemia	30	11	7.36E-04
Hepatitis B	39	11	1.14E-02
Endometrial cancer	19	11	1.27E-02
MicroRNAs in cancer	47	10	1.26E-07
Glycosaminoglycan biosynthesis - chondroitin sulfate / dermatan sulfate	7	10	2.17E-07
Gap junction	34	10	5.78E-07
Cell cycle	29	10	9.69E-05
Adherens junction	38	10	2.19E-04
Bacterial invasion of epithelial cells	30	10	2.45E-04
Viral carcinogenesis	62	10	3.49E-04
Nicotine addiction	24	10	4.40E-04
Adrenergic signaling in cardiomyocytes	52	10	1.12E-03
Glycosphingolipid biosynthesis - ganglio series	5	10	2.76E-03
RNA transport	32	9	3.42E-04
Small cell lung cancer	24	9	6.94E-04
Prolactin signaling pathway	23	9	9.83E-04
Dopaminergic synapse	57	9	4.96E-03
Type II diabetes mellitus	9	9	5.05E-03
HIF-1 signaling pathway	33	9	6.23E-03
Pancreatic cancer	25	9	1.11E-02
Glycosphingolipid biosynthesis - lacto and neolacto series	11	8	2.17E-14
Glutamatergic synapse	43	8	1.55E-04
Arrhythmogenic right ventricular cardiomyopathy (ARVC)	30	8	2.14E-04
Melanogenesis	36	8	4.00E-04
Protein digestion and absorption	12	8	7.88E-04
Nucleotide excision repair	9	8	1.38E-03
Sphingolipid signaling pathway	29	8	2.40E-03
Pyrimidine metabolism	11	8	1.18E-02
Ovarian steroidogenesis	4	8	1.38E-02
Inflammatory mediator regulation of TRP channels	32	8	2.56E-02
Cell adhesion molecules (CAMs)	39	7	1.16E-03
Viral myocarditis	10	7	1.29E-02
Choline metabolism in cancer	40	7	1.38E-02
Insulin signaling pathway	40	7	2.53E-02
Other types of O-glycan biosynthesis	12	6	4.55E-09
Axon guidance	56	6	9.34E-06
Retrograde endocannabinoid signaling	34	6	3.32E-03
Oxytocin signaling pathway	49	6	3.37E-03
Hedgehog signaling pathway	12	6	5.89E-03
Progesterone-mediated oocyte maturation	22	6	6.18E-03
Alcoholism	28	6	9.88E-03
Prion diseases	1	5	1.48E-45
Allograft rejection	7	5	1.32E-06
Folate biosynthesis	3	5	1.57E-06
One carbon pool by folate	4	5	2.33E-05
Synaptic vesicle cycle	14	5	8.04E-05
Inositol phosphate metabolism	19	5	1.54E-04
Endocytosis	46	5	2.66E-04
2-Oxocarboxylic acid metabolism	4	5	8.51E-04
Central carbon metabolism in cancer	12	5	1.03E-03
Sphingolipid metabolism	10	5	1.05E-03
SNARE interactions in vesicular transport	12	5	1.36E-03
NF-kappa B signaling pathway	9	5	1.56E-03
Circadian rhythm	11	5	1.62E-03

Glycerophospholipid metabolism	18	5	2.68E-03
Glycosphingolipid biosynthesis - globo series	4	5	1.08E-02
Valine, leucine and isoleucine degradation	10	5	1.12E-02
Phenylalanine, tyrosine and tryptophan biosynthesis	3	5	1.24E-02
Colorectal cancer	11	5	1.38E-02
Aldosterone-regulated sodium reabsorption	15	5	2.08E-02
Insulin secretion	20	5	2.13E-02
HTLV-I infection	41	5	4.13E-02
Thyroid hormone synthesis	19	4	7.87E-12
Fatty acid degradation	5	4	6.02E-08
Nicotinate and nicotinamide metabolism	6	4	5.32E-05
N-Glycan biosynthesis	15	4	2.53E-04
Maturity onset diabetes of the young	6	4	2.67E-04
Protein processing in endoplasmic reticulum	31	4	3.61E-04
Fatty acid metabolism	11	4	4.75E-04
Cholinergic synapse	23	4	1.35E-03
Sulfur metabolism	3	4	2.38E-03
Calcium signaling pathway	37	4	2.96E-03
Degradation of aromatic compounds	1	4	4.12E-03
Salivary secretion	18	4	4.97E-03
Pancreatic secretion	22	4	5.59E-03
Primary bile acid biosynthesis	3	4	1.21E-02
Herpes simplex infection	7	4	1.38E-02
T cell receptor signaling pathway	29	4	1.49E-02
Tight junction	21	4	1.92E-02
Leukocyte transendothelial migration	16	4	2.21E-02
Alanine, aspartate and glutamate metabolism	9	4	2.26E-02
Mismatch repair	1	4	3.09E-02
Steroid hormone biosynthesis	8	3	1.21E-15
Porphyrin and chlorophyll metabolism	11	3	6.73E-05
Other glycan degradation	1	3	1.37E-04
Biosynthesis of unsaturated fatty acids	3	3	3.54E-04
Serotonergic synapse	18	3	5.44E-04
Thiamine metabolism	1	3	7.39E-04
Fanconi anemia pathway	8	3	8.22E-04
Platelet activation	24	3	9.14E-04
Retinol metabolism	14	3	2.33E-03
Apoptosis	9	3	3.82E-03
Acute myeloid leukemia	10	3	4.86E-03
Endocrine and other factor-regulated calcium reabsorption	9	3	5.18E-03
Adipocytokine signaling pathway	17	3	6.39E-03
Cardiac muscle contraction	13	3	7.38E-03
GnRH signaling pathway	21	3	8.54E-03
D-Glutamine and D-glutamate metabolism	1	3	8.75E-03
Propanoate metabolism	3	3	1.01E-02
Bladder cancer	11	3	1.50E-02
Vascular smooth muscle contraction	14	3	1.80E-02
Vitamin digestion and absorption	3	3	1.99E-02
RNA degradation	16	3	2.31E-02
Toll-like receptor signaling pathway	4	3	2.50E-02
Chagas disease (American trypanosomiasis)	13	3	2.70E-02
Biotin metabolism	1	2	2.34E-11
Vasopressin-regulated water reabsorption	14	2	1.14E-07
Taste transduction	2	2	2.16E-05
Antigen processing and presentation	9	2	2.35E-05
Shigellosis	9	2	4.61E-05
Drug metabolism - cytochrome P450	8	2	8.19E-05
Hematopoietic cell lineage	7	2	1.85E-04
Pantothenate and CoA biosynthesis	3	2	3.26E-04
Steroid biosynthesis	3	2	3.95E-04
Ascorbate and aldarate metabolism	7	2	4.11E-04
Amyotrophic lateral sclerosis (ALS)	11	2	7.41E-04
Gastric acid secretion	12	2	8.58E-04
Arachidonic acid metabolism	6	2	8.99E-04
Autoimmune thyroid disease	4	2	9.67E-04
Metabolism of xenobiotics by cytochrome P450	3	2	1.38E-03
Cysteine and methionine metabolism	3	2	1.75E-03
Proteasome	3	2	2.07E-03
Pentose and glucuronate interconversions	7	2	3.51E-03
Glyoxylate and dicarboxylate metabolism	3	2	5.26E-03
Ribosome biogenesis in eukaryotes	5	2	5.62E-03
Salmonella infection	6	2	5.84E-03
Fc gamma R-mediated phagocytosis	11	2	7.03E-03
Drug metabolism - other enzymes	8	2	1.02E-02
Fc epsilon RI signaling pathway	13	2	1.27E-02
Notch signaling pathway	4	2	1.40E-02
Purine metabolism	4	2	1.51E-02
Pathogenic Escherichia coli infection	5	2	1.75E-02
Citrate cycle (TCA cycle)	4	2	2.53E-02
Starch and sucrose metabolism	8	2	3.19E-02
Graft-versus-host disease	5	2	3.89E-02
Asthma	4	2	4.11E-02
Tryptophan metabolism	2	2	4.53E-02
Taurine and hypotaurine metabolism	2	2	4.65E-02
Fatty acid elongation	2	1	1.72E-04
Terpenoid backbone biosynthesis	2	1	3.38E-04
Inflammatory bowel disease (IBD)	5	1	4.66E-04
B cell receptor signaling pathway	12	1	1.06E-03
Jak-STAT signaling pathway	9	1	1.24E-03
Renin-angiotensin system	2	1	1.36E-03
Bile secretion	6	1	2.45E-03
Ether lipid metabolism	2	1	2.70E-03
Non-alcoholic fatty liver disease (NAFLD)	10	1	6.85E-03
Glycosylphosphatidylinositol(GPI)-anchor biosynthesis	2	1	7.18E-03
Biosynthesis of amino acids	3	1	1.00E-02
Ribosome	1	1	1.18E-02
Peroxisome	7	1	1.23E-02
Neuroactive ligand-receptor interaction	13	1	1.36E-02
Osteoclast differentiation	16	1	1.61E-02
Leishmaniasis	4	1	1.99E-02
Fructose and mannose metabolism	2	1	2.12E-02
Phototransduction	2	1	2.19E-02
Phagosome	5	1	2.39E-02
VEGF signaling pathway	4	1	2.62E-02
Amino sugar and nucleotide sugar metabolism	1	1	2.75E-02
Systemic lupus erythematosus	1	1	2.77E-02
Riboflavin metabolism	2	1	2.97E-02
Base excision repair	1	1	2.98E-02
Cyanoamino acid metabolism	1	1	3.05E-02
Protein export	1	1	3.24E-02
Arginine and proline metabolism	2	1	3.35E-02

Epstein-Barr virus infection	9	1	3.48E-02
beta-Alanine metabolism	1	1	3.89E-02
Homologous recombination	2	1	3.90E-02
Pertussis	5	1	3.97E-02
Intestinal immune network for IgA production	3	1	4.21E-02
Ubiquinone and other terpenoid-quinone biosynthesis	1	1	4.23E-02
Glycolysis / Gluconeogenesis	2	1	4.49E-02
Spliceosome	3	1	4.63E-02
Alzheimer's disease	7	1	4.69E-02
Mineral absorption	4	1	4.90E-02

Table S4. Immune-related KEGG pathway analysis of HBM-derived exosomal miRNA

Term name	Number of Related gene	Number of miRNA	P-value
Cancer and Tumor Related Pathways			
Proteoglycans in cancer	109	25	< 0.05
Transcriptional misregulation in cancer	66	15	4.88E-12
Pathways in cancer	142	15	2.12E-14
MicroRNAs in cancer	45	8	1.77E-07
Central carbon metabolism in cancer	12	5	1.03E-03
Renal cell carcinoma	36	17	1.53E-05
Endometrial cancer	19	11	1.27E-02
Prostate cancer	45	14	4.32E-04
Chronic myeloid leukemia	30	11	7.36E-04
Basal cell carcinoma	17	16	2.59E-04
Melanoma	29	15	3.31E-05
Acute myeloid leukemia	10	3	4.86E-03
Non-small cell lung cancer	25	16	2.09E-03
Thyroid cancer	12	14	7.51E-05
Small cell lung cancer	24	9	6.94E-04
Colorectal cancer	11	5	1.38E-02
Bladder cancer	11	3	1.50E-02
Signaling Pathways			
Cell cycle	29	10	9.69E-05
Ras signaling pathway	101	15	9.41E-04
PI3K-Akt signaling pathway	149	19	3.45E-04
HIF-1 signaling pathway	33	9	6.23E-03
mTOR signaling pathway	30	19	1.86E-03
Hippo signaling pathway	64	14	4.95E-09
p53 signaling pathway	35	16	1.59E-05
Notch signaling pathway	4	2	1.40E-02
VEGF signaling pathway	4	1	2.62E-02
Pathogenic Microbial Infection			
Viral myocarditis	10	7	1.29E-02
HTLV-I infection	41	5	4.13E-02
Hepatitis B	39	11	1.14E-02
Viral carcinogenesis	62	10	3.49E-04
Epstein-Barr virus infection	9	1	3.48E-02
Herpes simplex infection	7	4	1.38E-02
Bacterial invasion of epithelial cells	30	10	2.45E-04
Pathogenic Escherichia coli infection	5	2	1.75E-02
Salmonella infection	6	2	5.84E-03
Shigellosis	9	2	4.61E-05
Chagas disease (American trypanosomiasis)	13	3	2.70E-02
Pertussis	5	1	3.97E-02
Amoebiasis	14	12	4.31E-06
Leishmaniasis	4	1	1.99E-02
Immune System and Inflammatory Disease			
Allograft rejection	7	5	1.32E-06
Graft-versus-host disease	5	2	3.89E-02
Toll-like receptor signaling pathway	4	3	2.50E-02
NF-kappa B signaling pathway	9	5	1.56E-03
Cytokine-cytokine receptor interaction	51	15	8.73E-08
Antigen processing and presentation	9	2	2.35E-05
Systemic lupus erythematosus	1	1	2.77E-02
Intestinal immune network for IgA production	3	1	4.21E-02
Renin-angiotensin system	2	1	1.36E-03
Non-alcoholic fatty liver disease (NAFLD)	10	1	6.85E-03
Inflammatory bowel disease (IBD)	5	1	4.66E-04
Others			
Hypertrophic cardiomyopathy (HCM)	5	1	4.66E-04
Prion diseases	1	5	1.48E-45
Dilated cardiomyopathy	34	17	4.45E-03
Arrhythmogenic right ventricular cardiomyopathy (ARVC)	30	8	2.14E-04
Fatty acid degradation	5	4	6.02E-08

Table S5. Total GO annotation of HBM-derived exosomal proteins

# Term ID	Term name	P-value
Gene Ontology: Cell Component		
GO:0005576	extracellular region	1.4E-155
GO:0031982	vesicle	8.2E-130
GO:0005829	cytosol	2.1E-36
GO:0031012	extracellular matrix	2.0E-13
GO:0005783	endoplasmic reticulum	1.2E-11
GO:0005886	plasma membrane	2.0E-10
GO:0005856	cytoskeleton	1.6E-04
GO:0005794	Golgi apparatus	2.6E-03
Gene Ontology: Molecular Function		
GO:0005198	structural molecule activity	8.0E-19
GO:0005102	signaling receptor binding	1.8E-15
GO:0019899	enzyme binding	2.9E-08
GO:0016787	hydrolase activity	1.2E-06
GO:0003824	catalytic activity	1.5E-05
GO:0005488	binding	2.1E-05
GO:0034987	immunoglobulin receptor binding	6.4E-05
GO:0045182	translation regulator activity	3.7E-04
GO:0016209	antioxidant activity	1.2E-03
GO:0023026	MHC class II protein complex binding	1.9E-03
GO:0019900	kinase binding	2.6E-03
GO:0003823	antigen binding	5.8E-03
GO:0030234	enzyme regulator activity	3.5E-02
Gene Ontology: Biological Process		
GO:0006412	translation	2.5E-23
GO:0006810	transport	3.3E-11
GO:0032502	developmental process	8.4E-10
GO:0048856	anatomical structure development	1.5E-09
GO:0009058	biosynthetic process	1.5E-07
GO:0065007	biological regulation	7.6E-06
GO:0006955	immune response	8.4E-06
GO:0008219	cell death	1.4E-05
GO:0030154	cell differentiation	1.8E-05
GO:0010628	positive regulation of gene expression	6.8E-05
GO:0023052	signaling	8.1E-04
GO:0007165	signal transduction	3.7E-03
GO:0007338	single fertilization	9.1E-03

Table S6. Immune-related GO annotation of HBM-derived exosomal protein

GO Category :Biological Process (immune related)		Number of proteins	-log10(p-value)
GO:0006952	Defense response	71	8.13
GO:0006909	Phagocytosis	27	7.95
GO:0002376	Immune system process	96	7.53
GO:0006959	Humoral immune response	26	6.97
GO:0006956	Complement activation	17	6.71
GO:0002252	Immune effector process	38	6.49
GO:0002253	Activation of immune response	27	5.82
GO:0045087	Innate immune response	43	5.38
GO:0006955	Immune response	68	5.07
GO:0006958	Complement activation, classical pathway	14	5.03
GO:0006911	Phagocytosis, engulfment	15	5.01
GO:0098542	Defense response to other organism	49	5.00
GO:0002682	Regulation of immune system process	57	4.83
GO:0050776	Regulation of immune response	41	4.63
GO:0034097	Response to cytokine	41	4.53
GO:0006954	Inflammatory response	37	4.23
GO:0009617	Response to bacterium	36	4.11
GO:0002684	Positive regulation of immune system process	41	3.91
GO:0002443	Leukocyte mediated immunity	26	3.87
GO:0016064	Immunoglobulin mediated immune response	17	3.85
GO:0019724	B cell mediated immunity	17	3.76
GO:0006910	Phagocytosis, recognition	12	3.39
GO:0002764	Immune response-regulating signaling pathway	26	3.06
GO:0002449	Lymphocyte mediated immunity	21	2.82
GO:0050851	Antigen receptor-mediated signaling pathway	17	2.59
GO:0071353	Cellular response to interleukin-4	7	2.55
GO:0001667	Ameboidal-type cell migration	23	2.48
GO:0042742	Defense response to bacterium	21	2.41
GO:0050853	B cell receptor signaling pathway	12	2.32
GO:0070670	Response to interleukin-4	7	2.28
GO:0002696	Positive regulation of leukocyte activation	23	2.26

Table S7. Total KEGG pathway analysis of HBM-derived exosomal protein

#Term ID	Term name	Number of protein	Protein list														P-value
hsa04145	Phagosome	14	C3	TLR2	THBS1	RAB7A	CD14	TUBA1B	TUBB	ACTB	RAB5B	CTSS	HLA-C	HLA-A	CD36	MRC1	8.71E-10
hsa04610	Complement and coagulation cascades	11	VTN	C3	C9	PLG	CLU	A2M	FGG	C4BPA	CFH	C4A	SERPINA1				5.15E-09
hsa05132	Salmonella infection	14	MAPK1	PFN1	GAPDH	TNFSF10	TLR2	RAB7A	CD14	HSP90AA1	ACTB	RAB5B	FLNA	HSP90AB1	RHOA	FLNB	3.56E-08
hsa05205	Proteoglycans in cancer	13	MAPK1	VTN	TLR2	THBS1	IQGAP1	FN1	ACTB	EZR	FLNA	HSPG2	RPS6	RHOA	FLNB		1.33E-07
hsa04612	Antigen processing and presentation	8	PDIA3	HSPA5	HSP90AA1	CTSS	HSP90AB1	HSPA1B	HLA-C	HLA-A							1.65E-06
hsa05152	Tuberculosis	11	MAPK1	CTSD	C3	TLR2	RAB7A	CD14	RAB5B	CTSS	HSPD1	RHOA	MRC1				1.92E-06
hsa04979	Cholesterol metabolism	7	APOB	APOA1	APOE	LPL	APOA4	APOA2	CD36								3.85E-06
hsa05130	Pathogenic Escherichia coli infection	10	MAPK1	MYH9	GAPDH	TNFSF10	NCL	TUBA1B	TUBB	ACTB	EZR	RHOA					3.63E-05
hsa04510	Focal adhesion	10	MAPK1	VTN	THBS1	TNC	EGF	FN1	ACTB	FLNA	RHOA	FLNB					5.28E-05
hsa04810	Regulation of actin cytoskeleton	10	MAPK1	MYH9	PFN1	EGF	IQGAP1	FN1	ACTB	EZR	GSN	RHOA					7.56E-05
hsa05150	Staphylococcus aureus infection	7	C3	ICAM1	PLG	FGG	CFH	KRT18	C4A								9.27E-05
hsa04512	ECM-receptor interaction	7	VTN	THBS1	TNC	FN1	HSPG2	NPNT	CD36								9.80E-05
hsa04144	Endocytosis	10	RAB11A	RAB7A	VPS35	KIF5B	RAB5B	HSPA1B	HLA-C	FOLR1	HLA-A	RHOA					2.00E-04
hsa05146	Amoebiasis	7	HSPB1	TLR2	C9	RAB7A	CD14	FN1	RAB5B								2.00E-04
hsa05133	Pertussis	6	MAPK1	C3	CD14	C4BPA	C4A	RHOA									3.60E-04
hsa05144	Malaria	5	TLR2	THBS1	CD81	ICAM1	CD36										4.80E-04
hsa03010	Ribosome	7	RPL3	RPL22	RPS6	RPL13A	RPS14	RPLP0	RPS19								7.60E-04
hsa05203	Viral carcinogenesis	8	MAPK1	C3	HIST2H2BE	GSN	HLA-C	HLA-A	RHOA	LYN							8.00E-04
hsa05134	Legionellosis	5	C3	TLR2	CD14	HSPA1B	HSPD1										9.00E-04
hsa04520	Adherens junction	5	MAPK1	IQGAP1	ACTB	RHOA	YES1										2.10E-03
hsa04151	PI3K-Akt signaling pathway	10	MAPK1	VTN	TLR2	THBS1	TNC	EGF	HSP90AA1	FN1	HSP90AB1	RPS6					2.50E-03
hsa03320	PPAR signaling pathway	5	APOA1	LPL	APOA2	CD36	ACSL1										3.10E-03
hsa04975	Fat digestion and absorption	4	APOB	APOA1	APOA4	CD36											3.40E-03
hsa04210	Apoptosis	6	MAPK1	CTSD	TNFSF10	TUBA1B	ACTB	CTSS									4.70E-03
hsa04915	Estrogen signaling pathway	6	MAPK1	CTSD	HSP90AA1	HSP90AB1	HSPA1B	KRT18									4.70E-03
hsa05169	Epstein-Barr virus infection	7	TLR2	ICAM1	PDIA3	HLA-C	HLA-A	LYN	VIM								5.20E-03
hsa05020	Prion disease	8	MAPK1	ATP5B	C9	KIF5B	HSPA5	TUBA1B	TUBB	HSPA1B							6.40E-03
hsa05416	Viral myocarditis	4	ICAM1	ACTB	HLA-C	HLA-A											7.90E-03
hsa04530	Tight junction	6	MYH9	TUBA1B	ACTB	EZR	RAB13	RHOA									8.90E-03
hsa04977	Vitamin digestion and absorption	3	APOB	APOA1	APOA4												8.90E-03
hsa05010	Alzheimer disease	9	MAPK1	GAPDH	APOE	ADAM10	ATP5B	KIF5B	LPL	TUBA1B	TUBB						8.90E-03
hsa05206	MicroRNAs in cancer	6	MAPK1	THBS1	TNC	EZR	RHOA	VIM									9.40E-03
hsa05164	Influenza A	6	MAPK1	TNFSF10	RAB11A	ICAM1	PLG	ACTB									1.06E-02
hsa04650	Natural killer cell mediated cytotoxicity	5	MAPK1	TNFSF10	ICAM1	HLA-C	HLA-A										1.58E-02
hsa04611	Platelet activation	5	MAPK1	FGG	ACTB	RHOA	LYN										1.59E-02
hsa05165	Human papillomavirus infection	8	MAPK1	VTN	THBS1	TNC	EGF	FN1	HLA-C	HLA-A							1.72E-02
hsa05167	Kaposi sarcoma-associated herpesvirus infection	6	MAPK1	C3	ICAM1	HLA-C	HLA-A	LYN									1.75E-02
hsa05418	Fluid shear stress and atherosclerosis	5	ICAM1	HSP90AA1	ACTB	HSP90AB1	RHOA										1.92E-02
hsa04015	Rap1 signaling pathway	6	MAPK1	PFN1	THBS1	EGF	ACTB	RHOA									2.40E-02
hsa04940	Type I diabetes mellitus	3	HLA-C	HSPD1	HLA-A												2.40E-02
hsa05219	Bladder cancer	3	MAPK1	THBS1	EGF												2.61E-02
hsa04540	Gap junction	4	MAPK1	EGF	TUBA1B	TUBB											2.63E-02
hsa04010	MAPK signaling pathway	7	MAPK1	HSPB1	EGF	CD14	FLNA	HSPA1B	FLNB								2.95E-02
hsa05131	Shigellosis	6	MAPK1	PFN1	C3	CD14	ACTB	RHOA									3.08E-02
hsa05322	Systemic lupus erythematosus	4	C3	C9	HIST2H2BE	C4A											3.08E-02
hsa05215	Prostate cancer	4	MAPK1	EGF	HSP90AA1	HSP90AB1											3.36E-02
hsa05142	Chagas disease	4	MAPK1	C3	TLR2	ACE											3.65E-02
hsa04620	Toll-like receptor signaling pathway	4	MAPK1	TLR2	CD14	TOLLIP											3.83E-02
hsa04141	Protein processing in endoplasmic reticulum	5	PDIA3	HSPA5	HSP90AA1	HSP90AB1	HSPA1B										3.97E-02
hsa04066	HIF-1 signaling pathway	4	MAPK1	GAPDH	EGF	RPS6											4.32E-02
hsa00061	Fatty acid biosynthesis	2	FASN	ACSL1													4.66E-02
hsa04670	Leukocyte transendothelial migration	4	ICAM1	ACTB	EZR	RHOA											4.66E-02
